# Perceptions of social media utilization among orthopaedic foot and ankle surgeons

**DOI:** 10.1186/s13047-023-00658-4

**Published:** 2023-09-08

**Authors:** Mehdi S. Salimy, Ankur S. Narain, Patrick B. Curtin, Eric C. Bellinger, Abhay R. Patel

**Affiliations:** 1 Department of Orthopedic Surgery, University of Massachusetts Memorial Medical Center, University of Massachusetts Chan Medical School, University Campus, 55 North Lake Avenue, Worcester, MA USA; 2 Department of Orthopaedic Surgery, Rush University Medical Center, Chicago, IL USA

**Keywords:** Foot, Ankle, Social media, Perception, Survey, Patient satisfaction

## Abstract

**Background:**

The growing social media presence in healthcare has provided physicians with new ways to engage with patients. However, foot and ankle orthopaedic surgeons have been found to underuse social media platforms despite their known benefits for patients and surgeons. Thus, this study sought to investigate the reasons for this phenomenon and to identify potential barriers to social media utilization in clinical practice.

**Methods:**

A 19-question survey was distributed to active attending physicians identified through the American Orthopaedic Foot & Ankle Society membership database. The survey included demographic, practice characteristics, and social media use questions assessed by a 5-point Likert scale. Logistic regression was used to identify predictors of positive attitudes toward social media.

**Results:**

Fifty-eight surgeons were included. Most respondents were male (*n* = 43, 74.1%), in private practice (*n* = 31, 53.5%), and described their practice to be greater than 51% elective procedures (*n* = 46, 79.4%). The average years in practice was 14.8 years (standard deviation, SD: 10.0 years). A total of 32.8% (*n* = 19) of surgeons reported using social media as part of their clinical practice. Facebook (*n* = 19, 32.8%), a professional website or blog (*n* = 18, 31.0%), and LinkedIn (*n* = 15, 25.9%) were the most used platforms–primarily for practice marketing or brand development (*n* = 19, 32.8%). A total of 58.6% (*n* = 34) of surgeons reported they did not use social media. The primary reasons were the time commitment (*n* = 31, 53.5%), concerns about obscuring professional boundaries (*n* = 22, 37.9%), and concerns regarding confidentiality (*n* = 11, 19.0%). Many surgeons reported that social media positively influences foot and ankle surgery (*n* = 23, 39.7%), although no individual predictors for these views could be identified.

**Conclusions:**

Foot and ankle orthopaedic surgeons tended to view social media use positively, but the time investment and concerns over professionalism and confidentiality pose challenges to its use. Given the influence of a surgeon’s social media identity on patient satisfaction and practice building, efforts should be made to streamline social media use for foot and ankle surgeons to establish their online presence.

**Level of evidence:**

Level IV, cross-sectional study.

**Supplementary Information:**

The online version contains supplementary material available at 10.1186/s13047-023-00658-4.

## Background

Advancements in the information technology era have ushered in new ways for patients and physicians to connect. For patients, the Internet provides an opportunity to better understand their health and supplement information from their healthcare provider. This further promotes patient autonomy [1], leading to greater patient satisfaction [2] and better health outcomes [3]. It is also a primary resource for patients when choosing a physician [4], with up to 26% of patients using physician rating websites when selecting their orthopaedic surgeon [5]. Accordingly, there has been increased social media utilization amongst academic and private practice orthopaedic surgeons to enhance their online presence through patient engagement. Social media can garner attention to peer-reviewed published research that may reach patients and lead to conversation in the clinical setting [6] while also being used as a tool to disseminate scholarly works among peers [7]. Additionally, surgeons can build their practice with a high return on investment from social media platforms such as Instagram and Facebook, which have demonstrated exponential increases in short-term practice revenue while providing more long-term linear growth [8]. Evidence also supports that an increased social media presence contributes to higher physician ratings on websites such as Google and Healthgrades, which are often crucial for initial patient recruitment while building a practice [9, 10].

Social networks differ in their monthly users, demographics, ease of use, and clinical utility in healthcare. Compared to surgeons, a much higher percentage of orthopaedic hospitals report having accounts on individual platforms, highlighting specialists’ underutilization of social media [11]. Moreover, there is variation in utilization between subspecialties. For example, there may be a greater need for social media representation in sports medicine due to a higher reported use among their patient population, mainly due to their younger age [5]. Studies have attempted to identify the most commonly used platforms by surgeons in each field as well as factors influencing their decision-making in choosing social media platforms to help explain these discrepancies, although no uniform predictors have been identified [12–17]. To better understand the effects of social media posts by orthopaedic surgeons, investigations on social platforms [18] and professional networks [19] have been performed, but these studies don’t fully characterize the impact of social media, much less address the potential consequences from the misuse of social media, which may be a deterrent for some surgeons. These often overlooked disadvantages include potential security breaches, inadvertent violations of privacy by sharing patient protected health information, inaccuracy of the information being shared, and questionable source credibility [20].

Still, some subspecialties are significantly underrepresented. When examining the top 100 social media influencers within orthopaedics on Twitter, 78% were identified as orthopaedic surgeons, with only 6% specializing in foot and ankle surgery [21]. A recent study demonstrated this low rate of social media utilization to be prevalent across all outlets without clearly identifiable causes or explanations for such [22]. Therefore, the primary purpose of this study was to identify the underlying reasons for the underuse of social media among foot and ankle orthopaedic surgeons and to identify potential barriers these surgeons face when considering establishing a social media presence for their clinical practice.

## Methods

### Identification of surgeons

Our study was exempt from Institutional Review Board approval at our institution. The senior author is a member of the American Orthopaedic Foot & Ankle Society and had access to the membership list. Active members were identified by the presence of an available email address in the database. Only attending physicians, denoted by either candidate, associate, or senior status, were considered for the purposes of this study. Members identified as fellows or residents were not included, as both are still in their surgical training and do not have independent practices. Inactive members were excluded.

A 19-question survey was created and distributed via e-mail from the American Orthopaedic Foot & Ankle Society to 744 eligible foot and ankle surgeons meeting the inclusion criteria. The e-mail described the context of the study and the voluntary nature of the survey. A six-month collection period was provided from June 2021 until November 2021, after which all archived responses were examined for completeness and analyzed. Responses were automatically captured upon completion and stored on an encrypted drive. The survey consisted of eight demographic and practice-specific questions, including age, gender, years in practice, practice setting (full-time academic vs. part-time clinical faculty/affiliated with university vs. private practice vs. other), practice demographics (urban vs. suburban vs. rural), practice geographic region (West vs. Midwest vs. South vs. Northeast), percentage of practice comprised of elective cases, and to what extent the surgeon was using social media as part of their clinical practice (Supplemental Fig. 1). Certain portions of the survey, such as platform utilization, allowed respondents to select multiple responses. Only fully completed responses were included in the final analysis.

### Social media use

The next series of survey questions consisted of four questions assessing social media use. This included identification of which platforms were being utilized (Facebook, Twitter, Instagram, Snapchat, LinkedIn, ResearchGate, YouTube, professional website or blog, TikTok, and/or Foursquare), reasoning for current or future use of social media in practice (practice marketing and brand development, patient education, networking with medical colleagues, dissemination of research or other academic works, and/or other), reasoning for not using social media in practice (concerns regarding confidentiality of patient health information, too much of a time commitment for an effective social media presence, worries about negative posts by patients on social media platform affecting practice, fear that social media use will obscure boundaries between professional and personal relationships with patients, and/or other), and whether surgeons who used social media had separate professional and personal accounts.

### Perceptions of social media in foot and ankle surgery

The final seven questions followed a 5-point Likert scale design to gain insight into foot and ankle surgeons’ perceptions of social media specific to the field. These included the following statements: social media has an overall positive influence on the field of foot and ankle surgery, social media use by foot and ankle surgeons worsens the image of the field, social media use by foot and ankle surgeons aids in the dissemination of new research and techniques, it is common for patients to look up their surgeons on social media to learn information about them and their practice, patients prefer to have a surgeon with an active social media presence, having a social media presence will contribute to the growth of my practice by attracting more patients, guidelines should be provided by a governing body (i.e. American Academy of Orthopaedic Surgeons, American Orthopaedic Foot & Ankle Society) detailing the appropriate use of social media for foot and ankle surgeons.

### Surgeon demographics

The final cohort consisted of 58 foot and ankle surgeons (7.8% response rate) (Table 1). Most respondents were between ages 41–50 years old (*n* = 21, 36.2%) and were male (*n* = 43, 74.1%). Notably, only 25.9% of respondents were under 40 years old. The average number of years in practice was 14.8 years (standard deviation, SD: 10.0 years) and most surgeons reported working in private practice (*n* = 31, 53.5%). Respondents were nearly even in describing their practice location as either suburban (*n* = 28, 48.3%) or urban (*n* = 27, 46.6%) and represented each of the four regions equally (West: *n* = 15, 25.9%; Midwest: *n* = 15, 25.9%; South: *n* = 14, 24.1%; Northeast: *n* = 14, 24.1%). Most surgeons described their practice as greater than 51% elective procedures (*n* = 46, 79.4%).

**Table 1:** Demographics of foot and ankle surgeon respondents

	***N* = 58**
**Characteristic**	**% (n)**
Age	
30 years or under	0% (0)
31–40 years	25.9% (15)
41–50 years	36.2% (21)
51–60 years	31.0% (18)
61 years or older	6.9% (4)
Gender	
Male	74.1% (43)
Female	25.9% (15)
Years in Practice (mean (SD))	14.8 (10.0)
Practice Setting	
Full-time academic	24.1% (14)
Part-time clinical faculty	13.8% (8)
Private practice	53.5% (31)
Other	8.6% (5)
Practice Location	
Urban	46.6% (27)
Suburban	48.3% (28)
Rural	5.1% (3)
Practice Region	
West	25.9% (15)
Midwest	25.9% (15)
South	24.1% (14)
Northeast	24.1% (14)
Elective Case Percentage	
Less than 25%	3.4% (2)
26–50%	17.2% (10)
51–75%	32.8% (19)
Greater than 75%	46.6% (27)

### Statistical methods

Descriptive statistics were represented as absolute numbers and corresponding percentages when appropriate. Continuous variables were reported as the mean and standard deviation. A logistic regression was performed to determine which predictor variables were associated with a positive attitude toward social media use in the field of foot and ankle surgery. Variables were only included if they demonstrated significance in univariate analysis. Statistical significance was defined as *p* < 0.05. All analyses were performed using STATA/MP 13.1 for Mac (StataCorp, College Station, TX).

## Results

### Surgeons and reported social media use

Of the 58 respondents, 32.8% (*n* = 19) reported using social media within their clinical practice (Table 2). The most used platforms were Facebook (*n* = 19, 32.8%), a professional website or blog (*n* = 18, 31.0%), and LinkedIn (*n* = 15, 25.9%). Marketing for practice and brand development was reported as the most common reason for using social media by 32.8% of surgeons, followed by reasons other than marketing, patient education, or networking (*n* = 15, 25.9%). More than half of surgeons reported time commitment as the reason for not using social media (*n* = 31, 53.5%), followed by concerns of obscuring professional boundaries (*n* = 22, 37.9%).

**Table 2:** Social media use among surgeons

	***N* = 58**
**Characteristic**	**% (n)**
Social Media Use in Practice	
Yes	32.8% (19)
No	58.6% (34)
Developing social media presence	8.6% (5)
Social Media Platform^a^	
Facebook	32.8% (19)
Professional Website/Blog	31.0% (18)
LinkedIn	25.9% (15)
Twitter	24.4% (14)
Instagram	19.0% (11)
YouTube	13.8% (8)
ResearchGate	10.3% (6)
Reasons for Using Social Media^b^	
Practice Marketing/Brand Development	32.8% (19)
Other	25.9% (15)
Patient Education	24.4% (14)
Networking	19.0% (11)
Reasons for Not Using Social Media^b^	
Time Commitment	53.5% (31)
Obscuring of Professional Boundaries	37.9% (22)
Concern Regarding Confidentiality	19.0% (11)
Effect of Negative Posts on Practice	15.5% (9)
Other	6.9% (4)

^a^Snapchat, TikTok, and Foursquare were each reported by 0% of surgeons

^b^Dissemination of Research/Academic Works was reported by 0% of surgeons

### How surgeons perceive social media in practice

Surgeons tended to positively perceive social media in foot and ankle surgery (Table 3). Most strongly agreed that patients look up their surgeons online through various social media platforms to learn more about them and their practice (*n* = 20, 35.0%) and agreed that social media could facilitate dissemination of research (*n* = 25, 43.1%), can help grow a practice by attracting more patients (*n* = 21, 36.2%), and that a governing body should outline social media guidelines for surgeons (*n* = 27, 46.6%). A large portion of respondents were neutral about the remaining questions. A logistic regression model with variables that demonstrated significance from the univariate analysis was utilized to determine predictors of having a positive attitude towards social media use in foot and ankle. However, none of the six demographic variables were significant (Table 4).

**Table 3:** Perceptions of social media in foot and ankle surgery

Survey Prompt	Strongly Agree	Agree	Neutral	Disagree	Strongly Disagree
*Social media has an overall positive influence on the field of foot and ankle surgery*	19.0%	20.7%	**43.1%**	10.3%	6.9%
*Social media use by foot and ankle surgeons worsens the image of the field*	2.0%	10.3%	**32.8%**	**32.8%**	22.4%
*Social media use by foot and ankle surgeons aids in the dissemination of new research and techniques*	12.0%	**43.1%**	31.0%	8.6%	5.2%
*It is common for patients to look up their surgeons on social media to learn information about them and their practice*	**35.0%**	33.3%	28.1%	3.5%	0%
*Patients prefer to have a surgeon with an active social media presence*	7.0%	12.1%	**43.1%**	34.5%	3.5%
*Having a social media presence will contribute to the growth of my practice by attracting more patients*	17.0%	**36.2%**	31.0%	12.1%	3.5%
*Guidelines should be provided by a governing body (i.e. American Academy of Orthopaedic Surgeons, American Orthopaedic Foot & Ankle Society) detailing the appropriate use of social media for foot and ankle surgeons*	14.0%	**46.6%**	20.7%	8.6%	10.3%

**Table 4:** Logistic regression analyzing positive attitude towards social media

Predictor	Coefficient	95% CI	*P*-Value
Age > 50 years	-0.22	-1.31–0.87	0.69
Female Gender	-1.25	-2.65–0.15	0.08
Years in Practice > 15	-0.38	-1.45–0.68	0.48
Private Practice	-0.37	-1.43–0.68	0.49
Urban Practice Setting	-0.50	-1.57–0.57	0.36
Elective Cases > 75%	-0.21	-1.26–0.85	0.70

*95% CI* 95% Confidence Interval

## Discussion

The current study found that most surgeons had a positive perception of social media in foot and ankle surgery, although there is a discordance between perceptions of social media and its utilization in practice. Despite most respondents believing that a social media presence could grow their practice and attract new patients, 58.6% of respondents were not using social media at the time of their survey. This is consistent with previous studies that have shown physicians value social media for sharing information with patients and colleagues [23], practice building [8], enhanced online reputation [15], and further highlights the underutilization of social media among foot and ankle surgeons [5, 22]. According to foot and ankle surgeons, not having enough time to dedicate to maintaining their social media accounts and concerns over professional boundaries and patient privacy may explain their hesitance in establishing an online presence.

Healthcare in the United States has rapidly evolved in the last few decades with the concurrent expansion of the Internet. The structure of the healthcare system is reliant upon complex interrelations between providers, patients, and systems that finance care. Consequently, it is crucial to understand factors that influence these pillars. Two such factors, information technology and the concept of the patient as a consumer influenced by social media and nontraditional channels of information, have been found to produce the most change, although their role in delivering value-based care is not entirely understood [24]. The adaptation to information technology can be seen from the results of a recent survey across all hospital types from the American Hospital Association that showed that over 95% of hospitals have certified electronic health record technology [25]. Social media in healthcare settings, while growing in fields such as orthopaedic surgery [26], continues to lag behind.

Although there has been growth in the field overall, certain subspecialties suffer from underutilization of social media. A study by Curry et al. surveying new patients to an orthopaedic clinic, discovered that patients seeing a foot and ankle specialist utilized social networking sites the most (59.7%) of out five subspecialties [5]. They also concluded that age was the biggest indicator predicting social media use, with older patients being less likely to use social media [5]. Interestingly, we were not able to identify any predictors for the perception of social media use in the field of foot and ankle amongst surgeons. This suggests that there is no one profile of an orthopaedic foot and ankle surgeon who is likely to have a positive attitude towards social media use in their practice. Rather, it seems that attitudes towards social media are largely dependent on personal experience and beliefs about its potential benefits. In addition, a recent study by Garofolo-Gonzalez et al. revealed that foot and ankle surgeons were only using 30% of social media platforms available to them and a low social media index across the platforms [22]. Our study reinforces underuse among foot and ankle surgeons, with only 32.8% of respondents stating that they use social media in practice and 8.6% stating they are in the process of forming a social media identity. We also found similar reports of the platforms utilized, with Facebook, a professional or group practice website, and LinkedIn being the three most used [22]. Social media has been viewed as a powerful tool for practice building [2], and our results further supported this view as brand development and practice marketing was the most common reason for social media utilization.

It is worth nothing that much of the information shared on social media is not reviewed by a regulatory group and subsequently is of poor quality [27]. There is also considerable variability in the number of orthopaedic websites presenting high-quality information ranging from 3 to 44% [28]. Most foot and ankle respondents from our study believed that guidelines outlining the appropriate use of social media, and thus standards of quality information distributed by surgeons, should be established by a governing organization. The American Academy of Orthopaedic Surgeons has provided an instructional course on the appropriate utilization of blogs for orthopaedic surgeons [29], but the blogging best practices did not address commonly used social media platforms. Concerns over professionalism and patient privacy were the two commonly reported barriers to using social media for clinical practice. Guidelines for professional use of social media have been published by The American Medical Society [30] and some orthopaedic journals [31] but again are not specific to a platform or to the field of foot and ankle surgery. While generalized rules are certainly valuable, more specific details for specialists may prove to be more useful in developing an online identity. The most prominent barrier to establishing a social media presence was the time burden associated with its use, which is in agreement from previous findings that time commitment is the main reason why more physicians have yet to adopt social media clinically [32]. To maximize efficiency and dedicate their time elsewhere, surgeons may opt into third-party software or management teams to coordinate their social media image and presence. Nevertheless, surgeons will ultimately be held responsible for these sites so they must be aware of the individuals they choose to represent them digitally, as errors or misinformation could have serious negative consequences for the surgeon’s practice reputation.

Arguably, the most important role of a social media presence is to allow for patients to preview their physician prior to consultation. As physician ratings and patient comments are becoming more readily available online, it is increasingly important for physicians to manage their online reputations. Patients view these ratings as an opportunity to make an informed decision when choosing their physician, and it also provides doctors with insight into what patients may value. Physician knowledge [33], friendliness, and communicative skills affect overall evaluations, but factors beyond the physician’s immediate skills such as cleanliness of the practice and modernity of the medical equipment also impact reviews [34]. In foot and ankle surgery, surgeons tend to have a favorable rating and surgeon personality is a significant factor leading to a higher proportion of positive reviews [35]. Moreover, nearly a quarter of respondents from our study use social media for patient education which may aid their social image. Reconsidering the patient as a consumer in the current healthcare model, there is strong evidence to support establishing an online presence for surgeons. The implications of social media on patient satisfaction and practice building are apparent and more foot and ankle surgeons should consider utilizing social media platforms.

There are several limitations to our study that should be considered when interpreting our results. First, our sample size was relatively small partly due to the identification process of qualifying surgeons within the American Orthopaedic Foot & Ankle Society membership database. Even so, surveys were collected over an extended period of time (six months) to maximize the response rate. Furthermore, respondents represented a broad and mostly balanced distribution of clinical practices and settings, supporting the generalizability of our findings. Next, the survey was self-reported which is inherently subject to response bias. To mitigate these effects, the survey was designed using discrete questions using neutral wording to avoid implicit bias, and multiple options were provided per question to prevent skewing of the results. However, the 5-point Likert scale with a neutral option may explain the large portion of respondents that remained neutral regarding social media perceptions compared to a 4-point or 6-point scale in which participants must reveal agreement or disagreement with a statement. Alternatively, the neutral population may emphasize a lack of knowledge or experience with social media and its potential benefits in the field of foot and ankle from responding surgeons. A final consideration was that this study did not explore the reasoning as to why surgeons selected certain social media platforms over others. Ease of use and outreach, among others, may influence a surgeon’s decision to use social, but this level of stratification might minimize the generalizability of the results considering the high variability in personal experiences. Despite these limitations, our findings provide valuable insights into surgeon perceptions of social media in foot and ankle surgery.

## Conclusions

Most foot and ankle surgeons had a positive attitude toward social media and believed that it has the potential to grow a practice and aid in the communication of information for fellow clinicians and patient education. However, the perceived time commitment necessary to establish and maintain a social media presence was found to be a significant barrier to its use. Concerns of professionalism and patient confidentiality may potentially be addressed by the call for clear guidelines for specialists from professional associations such as the American Academy of Orthopaedic Surgeons. Future studies should explore the efficacy of implementing social media account managers on practice volume and outreach, as a dedicated social media team may be an investment for orthopaedic surgeons in delivering high quality care while expanding their practice.

### Supplementary Information

**Additional file 1: Supplemental Fig. 1.** This figure shows the survey that was provided to qualifying orthopaedic foot and ankle surgeons to assess social media utilization and perceptions of its use in practice.

## Data Availability

The datasets used and/or analyzed during the current study are available from the corresponding author on reasonable request. A copy of the survey distributed to qualifying surgeons has been provided as a supplemental figure to this text.
