# Supplementary material for: Perceptions of social media utilization among orthopaedic foot and ankle surgeons
Source: J Foot Ankle Res. 2023 Sep 8;16:58. doi: 10.1186/s13047-023-00658-4 (PMC10486077; doi:10.1186/s13047-023-00658-4)
Supplement: Supplementary file 1 — Additional file 1: Supplemental Fig. 1. This figure shows the survey that was provided to qualifying orthopaedic foot and ankle surgeons to assess social media utilization and perceptions of its use in practice. [file 13047_2023_658_MOESM1_ESM.pdf]

Social Media Utilization by Foot and Ankle Surgeons

Resize font:

The extent to which foot and ankle surgeons use social media for professional purposes has not been well studied. We have designed this anonymous survey to provide insights into why surgeons use social media within their practice, and to identify any potential barriers towards social media implementation.

Thank you!

Demographic and Practice Information

What is your age?
- ○ 30 years or under
- ○ 31-40 years
- ○ 41-50 years
- ○ 51-60 years
- ○ 61 years or older

reset

What is your gender?
- ○ Male
- ○ Female

reset

How many years have you been in practice? ________

How would you describe your practice setting?
- ○ Full-time Academic
- ○ Part-time clinical faculty (affiliated with university)
- ○ Private Practice
- ○ Other

reset

How would you describe the location of your practice?
- ○ Urban
- ○ Suburban
- ○ Rural

reset

What region of the country is your practice located in?
- ○ West
- ○ Midwest
- ○ South
- ○ Northeast

reset

What percentage of your practice is comprised of elective cases?
- ○ Less than 25%
- ○ 26-50%
- ○ 51-75%
- ○ Greater than 75%

reset

Do you use social media as part of your clinical practice?
- ○ Yes
- ○ No
- ○ I am currently in the process of developing a social media presence
- ○ I used social media prior, but no longer do so

reset

Social Media Use

What types of social media do you use in your clinical practice? (Mark all that apply)
- ☐ Facebook
- ☐ Twitter
- ☐ Instagram
- ☐ Snapchat
- ☐ LinkedIn
- ☐ ResearchGate
- ☐ YouTube
- ☐ Professional website or blog
- ☐ TikTok
- ☐ Foursquare

If you use or plan to use social media in your practice, what is your reason for doing so? (Mark all that apply)
- ☐ Practice marketing and brand development
- ☐ Patient education
- ☐ Networking with medical colleagues
- ☐ Dissemination of research or other academic works
- ☐ Other

If you do not use social media in your practice, what is your reason for not doing so? (Mark all that apply)
- ☐ I am concerned about the confidentiality of patient health information
- ☐ The time commitment for an effective social media presence is too high
- ☐ I am worried that negative posts by patients on social media platforms will affect my practice
- ☐ I am afraid that social media use will obscure the boundaries between professional and personal relationships with patients
- ☐ Other

If you use social media for both professional and personal reasons, do you have separate professional and personal accounts?
- ○ Yes
- ○ No
- ○ I do not use social media in my practice

reset

For the following questions, please rate your level of agreement with the provided statements

Social media has an overall positive influence on the field of foot and ankle surgery
- ○ Strongly agree
- ○ Agree
- ○ Neutral
- ○ Disagree
- ○ Strongly disagree

reset

The use of social media by foot and ankle surgeons worsens the image of the field
- ○ Strongly agree
- ○ Agree
- ○ Neutral
- ○ Disagree
- ○ Strongly disagree

reset

The use of social media by foot and ankle surgeons aids in the dissemination of new research and techniques
- ○ Strongly agree
- ○ Agree
- ○ Neutral
- ○ Disagree
- ○ Strongly disagree

reset

It is common for patients to look up their surgeons on social media to learn information about them and their practice.
- ○ Strongly agree
- ○ Agree
- ○ Neutral
- ○ Disagree
- ○ Strongly disagree

reset

Patients prefer to have a surgeon with an active social media presence
- ○ Strongly agree
- ○ Agree
- ○ Neutral
- ○ Disagree
- ○ Strongly disagree

reset

Having a social media presence will contribute to the growth of my practice by attracting more patients
- ○ Strongly agree
- ○ Agree
- ○ Neutral
- ○ Disagree
- ○ Strongly disagree

reset

Guidelines should be provided by a governing body (i.e. AAOS, AOFAS) detailing the appropriate use of social media for foot and ankle surgeons
- ○ Strongly agree
- ○ Agree
- ○ Neutral
- ○ Disagree
- ○ Strongly disagree

reset

Submit

Powered by REDCap
